# Engineering a Lactobacillus Lysine Riboswitch to Dynamically Control Metabolic Pathways for Lysine Production in *Corynebacterium glutamicum*

**DOI:** 10.3390/microorganisms12030606

**Published:** 2024-03-18

**Authors:** Qingwei Jiang, Feng Geng, Juan Shen, Ping Zhu, Zhaoxin Lu, Libang Zhou, Fengxia Lu

**Affiliations:** 1 College of Food Science and Technology, Nanjing Agricultural University, Nanjing 210095, China; 2 College of Pharmacy, Binzhou Medical University, Yantai 264003, China; gengfeng@bzmc.edu.cn

**Keywords:** gene expression, lysine riboswitch, dynamic control, metabolic pathway, *C. glutamicum*

## Abstract

Knock-out of genes of metabolic pathways is conventionally used in the metabolic engineering of microorganisms, but it is not applicable for genes of essential pathways. In order to avoid undesirable effects caused by gene deletion, it is attractive to develop riboswitches to dynamically control the metabolic pathways of microbial cell factories. In this regard, the aim of this study is to utilize the lysine riboswitch to control gene expressions of the biosynthetic pathways and by-pathways and thus improve lysine production in *Corynebacterium glutamicum*. To achieve this, a natural lysine riboswitch from *Lactobacillus plantarum* (LPRS) was first detected and then fused with *RFP* to test its functionality. After that, engineered lysine-activated (Lys-A) and lysine-repressed (Lys-R) riboswitches were successfully screened by dual genetic selection. Furthermore, the optimized A263 and R152 were applied to control the expression of aspartate kinase III and homoserine dehydrogenase in the lysine-producing strain *C. glutamicum* QW45, respectively. In contrast with QW45, the growth of the resulting A263-lysC mutant QW48 was similar to that of QW45; however, the growth of the resulting R357-hom mutant QW54 was slightly inhibited, indicating an inhibition of threonine biosynthesis caused by the riboswitch upon binding of intracellular lysine. Importantly, the lysine production of QW48 and QW54 was, respectively, 35% and 43% higher than that of the parent strain QW45, implying more metabolic flux directed into the lysine synthesis pathway. Finally, the engineered A263 and R357 were simultaneously applied to the same mutant QW55, which greatly improved lysine production. Thus, the approach demonstrated in this work could be principally used as a powerful tool to dynamically control any other undesired metabolic pathways.

## 1. Introduction

Riboswitches have been discovered in nature and are widely applied for regulating gene expression based on small-molecule-RNA interaction [1]. As is known, most natural riboswitches are found in the 5′ untranslated region (UTR) of an mRNA and consist of a sensor region (aptamer domain) and an expression platform. The aptamer domain could sense the target metabolite with a high binding affinity. The structure of the expression platform could be changed in response to ligand-induced conformational changes, which resulted in varied gene expression [2]. Until now, there have been more than 50 riboswitches validated by experiments. However, only three of them were involved in amino acid biosynthesis: lysine riboswitch, glycine riboswitch, and glutamine riboswitch [3,4]. There have been some successful evidence showing that engineering riboswitches is an effective method to control gene expression [5,6,7]. 

L-lysine is widely used for food and chemicals nowadays and is typically fermentatively produced with *Corynebacterium glutamicum* in the industry [8]. Considering the increasing demand for L-lysine, a continuous optimization of lysine-producing strains such as *C. glutamicum* has been made through various methods [9,10]. So far, there have been many successful-producing strains obtained by metabolic engineering. With these efforts, lysine production could be, for example, improved by: (1) removing allosteric feedback inhibition; (2) enhancing precursor supply; (3) enhancing cofactor (NADPH) generation; (4) dynamic control of the byproduct pathway [11,12,13]. However, it was still a big challenge to realize dynamic control of the biosynthesis of the enzymes. The toolbox for the control of gene expression was limited, which included, e.g., allosteric enzyme regulation, promoter replacement, and Shine-Dalgarno (SD) optimization. Thus, there were many significant efforts to overcome the bottlenecks by using RNA as a novel type of tool [14,15]. 

In *C. glutamicum*, several different riboswitches had been predicted to be located in the 5′-UTRs by using an improved RNAseq technique [16]. A flavin mononucleotide (FMN) riboswitch was discovered to be present upstream of the *ribM* gene in *C. glutamicum* [17]. Recently, a ydaO/kimA-type cyclic di-adenosine monophosphate (c-di-AMP) riboswitch was found to strictly regulate the expression of the cell wall peptidase gene *nlpC* in *C. glutamicum* [18]. Although riboswitch-like sequences of 5′-UTRs might be contained in some genes associated with the metabolism of amino acids (e.g., methionine, histidine) [16], it was still unknown whether natural lysine riboswitch existed in *C. glutamicum* or not. However, lysine riboswitches in *E. coli* and also *Bacillus subtilis* had been well studied, which were able to directly control the translation initiation of the corresponding mRNA as well as its stability simultaneously [19]. Interestingly, a similar phenomenon was found for the FMN riboswitch of *C. glutamicum*, this indicated the two riboswitches from different organisms function with the same mechanism [20]. 

**Figure 1:**
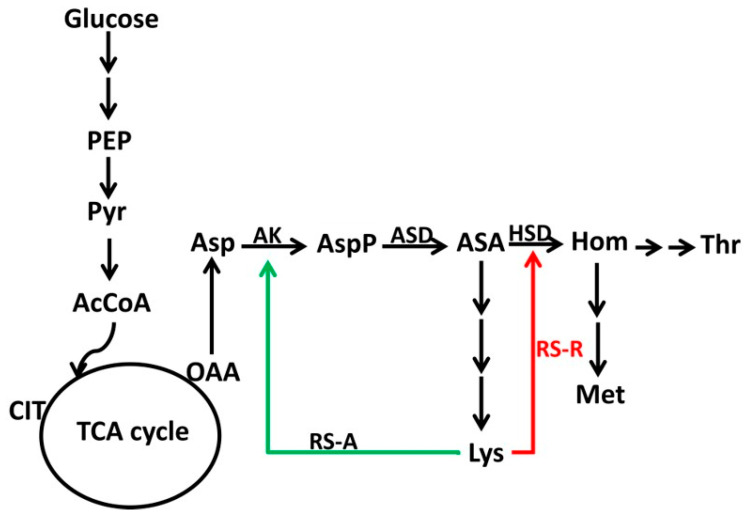
A simplified illustration of the lysine biosynthetic pathway in *C. glutamicum*. Abbreviation: AK, aspartate kinase (*lysC*); ASD, aspartyl semialdehyde dehydrogenase (*asd*); HSD, homoserine dehydrogenase (*hom*); RS-A, riboswitch-activated regulation; RS-R, riboswitch-repressed regulation.

In the aspartate metabolic pathway, aspartate kinase III (AK III, *lysC*) was considered the key enzyme of lysine biosynthesis in *C. glutamicum*. Previous studies proved that the expression and the activity of AK III could be regulated by lysine. Considering this, it was attractive to overexpress a mutant *lysC* gene to improve lysine production. On the other hand, the biosynthesis of threonine and methionine were both branch pathways of lysine biosynthesis deriving from aspartate semialdehyde (Figure 1). In *C. glutamicum*, homoserine dehydrogenase (HSD, *hom*), the key enzyme of threonine biosynthesis, could be feedback inhibited by threonine. It had been demonstrated that the *hom* single mutant HD-1 could get a yield of 10 g/L of lysine [21]. Furthermore, an allosteric homoserine dehydrogenase (HSD) was rationally designed to be inhibited by lysine [22]. Considering its importance to cell growth, it was also of great interest to improve lysine production by dynamically controlling the expression of the *hom* gene.

In previous work, a natural *E. coli* lysine riboswitch was applied to repress the expression of citrate synthase (*gltA*) in *C. glutamicum*, which resulted in a significant improvement in lysine production [23]. Furthermore, an engineered *E. coli* lysine riboswitch was generated to promote the excretion of lysine in *C. glutamicum* [24]. These studies suggested that riboswitches showed promise in metabolic engineering and synthetic biology. However, *E. coli* was not considered a food-grade bacterium. To our interest, lysine riboswitches, which were from safe bacteria, could be easy to accept and also apply in food fermentation. Furthermore, it was also meaningful to investigate more lysine riboswitches as useful tools for genetic regulatory circuits to simultaneously control several gene expressions. To address those, a natural lysine riboswitch from *Lactobacillus plantarum* was first identified and characterized in this study. Then, several engineered lysine riboswitches were screened and demonstrated. Finally, the selected lysine riboswitches were introduced into the chromosome of *C. glutamicum* to improve lysine production via the dynamic control of two different metabolic pathways. This work proved that more riboswitches could be widely used in *Corynebacterium* species for molecular biotechnology and industrial applications. Importantly, the results obtained in this study demonstrated that the aim of applying a biologically safe lysine riboswitch to dynamically control metabolic pathways was successfully achieved. 

## 2. Materials and Methods

### 2.1. Bacterial Strains and Media

The bacterial strains and plasmids utilized in this study are listed in Table S1. *E. coli* DH5α (Vazyme, Nanjing, China) was regularly used as the host for normal cloning and plasmid construction [25]. *C. glutamicum* ATCC 13032 was used as the starting strain for genetic modification. All *E. coli* strains were normally cultured at 37 °C in Luria-Bertani (LB) broth or on LB agar plates. The chemical Rich Defined Medium (RDM) was used to measure fluorescence [26]. Trypticase soy broth medium was used to culture *C. glutamicum* strains at 30 °C. When necessary, the media contained 100 µg/mL Ampicillin for *E. coli* or 25 µg/mL Kanamycin for *C. glutamicum*. Other chemicals were purchased from Sinopharm Chemical Reagent (Nanjing, China) or Nanjing Chemical Reagent (Jiangsu, China).

### 2.2. DNA Manipulation

The genomic DNA of *C. glutamicum* was extracted using standard protocols. DNA polymerase chain reaction (PCR), digestion, and ligation were carried out according to the manufacturer’s instructions (Takara, Dalian, China). The plasmid isolation and gel purification kits were both obtained from Vazyme (Nanjing, China). Oligonucleotides were synthesized by Genscript (Nanjing, China) and are listed in Table S2. DNA sequencing was carried out by Genewiz (Suzhou, China). The transformation of *E. coli* or *C. glutamicum* was performed as described before [27].

### 2.3. Identification of Lysine Riboswitches in Lb. plantarum

Previous studies have shown that the lysine riboswitch is normally located upstream of the *lysC* gene [28,29]. To find out whether there were putative lysine riboswitches in *C. glutamicum* and also *Lb. plantarum*, the web-application Riboswitch Scanner [30] was used to detect lysine riboswitch from genomic sequences. Meanwhile, the coding sequences of lysine riboswitch from *Bacillus subtilis* were blasted against the *C. glutamicum* 13032, and also *Lb. plantarum* WCFS1 genome, respectively. Subsequently, the secondary structure of the potential lysine riboswitch was predicted by RNAfold [31] and visualized using RNAcanvas [32]. 

### 2.4. Construction of Plasmids

Standard protocols were carried out for the construction of plasmids according to the standard methods. For the construction of lysine riboswitch-RFP, the lysine riboswitch (LPRS) was amplified with primers LPRS-F and LPRS-R from *Lb. plantarum* WCFS1 genomic DNA (Table S2). Then this fragment was fused with a fragment amplified by BR-F and BR-R from pJ-RFP harboring the synthetic promoter BBA_J23100 to generate pLPRS-RFP. To insert the LPRS into the chromosome of *C. glutamicum*, plasmid pK-LPRSA-lysC was constructed based on the suicide vector pK18mobsacB [27]. Four fragments (the upstream of the *lysC* gene, including the native promoter of *lysC*, LPRS, the native *lysC* gene, and the backbone of pK18mobsacB) were amplified by PCR, respectively (Table S2). Then, pK-LPRSA-lysC was generated by fusing these fragments using the ClonExpress MultiS One Step Cloning Kit (Vazyme, Nanjing, China). A similar procedure was performed to construct the plasmid pK-LPRSR-hom.

### 2.5. Fluorescence Measurement

The pLPRS-mRFP was transformed into *E. coli* DH5α to test the functionality of the lysine riboswitch. Single colonies were inoculated into 2 mL of RDM and grown overnight. The overnight cultures were diluted 100 times in 1.5 mL of fresh RDM supplemented with an appropriate lysine concentration. After being cultured for 8 h, 300 μL of cells were harvested and washed using phosphate-buffered saline (PBS) and then resuspended in 300 μL of fresh PBS. To make three replicates, 100 μL of resuspension was transferred into a 96-well microplate. *RFP* fluorescence (excitation 535 nm and emission 620 nm) was measured using the Spark^TM^ Multimode Microplate Reader (Tecan, Grödig, Austria). The optical density of *E. coli* cells was detected at 600 nm. Background fluorescence obtained from wells filled with PBS was subtracted for each measurement. Relative fluorescence values were normalized by the corresponding cell density (OD_600_) values [33]. 

### 2.6. Screening Engineered Lb. plantarum Lysine Riboswitches

To obtain the engineered lysine riboswitches, the dual genetic selection procedure was performed following previous work with minor modifications. First, a riboswitch library was constructed using primers RSlib-F and RSlib-R with pLPRS-tetA-RFP as a template. Then, the library cells were cultured in an RDM-Amp medium supplemented with 100 μM lysine. After three rounds of the dual genetic selection, the colonies that could grow on RDM-Amp plates supplemented with 30 μg/mL tetracycline with lysine (100 μM) but not without lysine were considered to have engineered lysine-activated riboswitches. On the other hand, after dual genetic selection, the cells that survived on RDM-Amp plates supplemented with 0.3 mM NiCl_2_ with lysine (100 μM) but not without lysine were considered to be engineered lysine-repressed riboswitches. The generated colonies were randomly selected for sequencing. Finally, the sequenced cells were cultivated in an RDM-Amp medium with an additional 100 μM lysine to measure the fluorescence of RFP. The measurements were accomplished in biological triplicates.

### 2.7. Construction of C. glutamicum Lysine Riboswitch-Controlled Mutants

Lysine-producing *C. glutamicum* QW45, which carries mutations of *lysC* (Q298G), was first constructed according to a previous report [34]. Plasmid pK-LPRSA-lysC, which was expected to replace the chromosomal *lysC* gene with lysine-activated riboswitch-lysC, was used to generate strain *C. glutamicum* QW48. Similarly, plasmid pK18-LPRSR-hom was used to create strain *C. glutamicum* QW53, and an engineered lysine-repressed riboswitch was inserted into the upstream of the *hom* gene in the genome. *C. glutamicum* electro-transformation was carried out following a previous report [27]. After two rounds of homologous recombination, the mutant *C. glutamicum* strains were obtained by screening and finally verified by DNA sequencing (Genewiz, Suzhou, China). The same strategy was further performed to generate mutant QW55, in which both *lysC* and *hom* were controlled by engineered lysine riboswitches.

### 2.8. Enzyme Activity Assay

To measure the enzyme activity, *C. glutamicum* strains were cultivated in 30 mL of Defined Minimal Medium (DMM) [11]. Cells were harvested at an OD of 4 by centrifugation (10,000 rpm, 4 °C, 10 min) and washed with 10 mL of 0.9% NaCl. Cell pellets were resuspended in 1 mL of 100 mM Tris-HCl/100 mM KCl buffer (pH 8.0). The cells were then disrupted by sonication for 8 min in an ice bath. Cell debris was removed by centrifugation (12,000 rpm, 4 °C, 10 min), and the supernatant was immediately used for the enzyme assay. Total protein concentrations were measured using the Bradford assay [35]. The specific activity of aspartate kinase and homoserine dehydrogenase was measured separately, as described previously [22,36]. 

### 2.9. Fermentation and Analytical Methods

Batch fermentations of *C. glutamicum* strains were carried out in DMM in a shake flask as described before [11]. Seed cultures were prepared in 100-mL Erlenmeyer flasks containing 20 mL of DMM and cultured at 30 °C with 230 rpm in a shaking incubator [34]. After reaching the exponential growth phase, the seed cells were harvested and washed twice with PBS. The pre-prepared cells were transformed into fresh DMM to achieve an initial optical density of 0.05. During the fermentation processes, the pH was detected and controlled at 7.2 when necessary. Cell growth was determined by measuring the OD_600_ (*E. coli*) or OD_660_ (*C. glutamicum*) after dilution with 0.1 M HCl with a UV spectrophotometer or using a Spark^TM^ Multimode Microplate Reader (Tecan, Grödig, Austria). The concentration of glucose was measured using an SBA-40D biosensor analyzer (Institute of Biology of Shandong Province Academy of Sciences, Shandong, China). The extracellular lysine concentration was measured using the ninhydrin method and HPLC (Shimadzu, Kyoto, Japan). 

### 2.10. Statistics Analysis

All the measurements were performed in biological triplicates. Data were analyzed by one-way analysis of variance and Dunnett’s multiple comparison test was performed to determine the significant differences. A value of *p* < 0.05 was considered significant. The results are presented as means ± standard deviation of triplicate measurements.

## 3. Results

### 3.1. A Putative Lactobacillus Lysine Riboswitch Controls a Lysine Transport Protein

Previous studies have shown that lysine riboswitch is always located in the upstream region of the *lysC* gene in most microorganisms. To investigate whether a native lysine riboswitch existed in *C. glutamicum*, the whole genome sequences were first analyzed by a Riboswitch Scanner. Interestingly, some common riboswitches, such as FMN, TPP, and SAM riboswitches, were all detected. However, no amino acid riboswitches were predicted in *C. glutamicum* 13032 (Table S3). In order to confirm this result, the regulatory region (242 bp) of the *lysC* gene between a hypothetical protein (cg0305) and the *lysC* gene (aspartate kinase, cg0306) in *C. glutamicum* was uploaded onto Riboswitch Finder. Unfortunately, the sequences did not match any discovered riboswitches. Importantly, previous work indicated that the transcriptional start site (TSS) of the *lysC* gene was located 36 bp upstream of the start codon (GTG) [37]. However, a typical aptamer of lysine riboswitch is as long as 170 bp [3], thus no native lysine riboswitch could be located in this short region in *C. glutamicum*. Therefore, the exogenous lysine riboswitch was considered to be introduced into *C. glutamicum* in the following.

**Figure 2:**
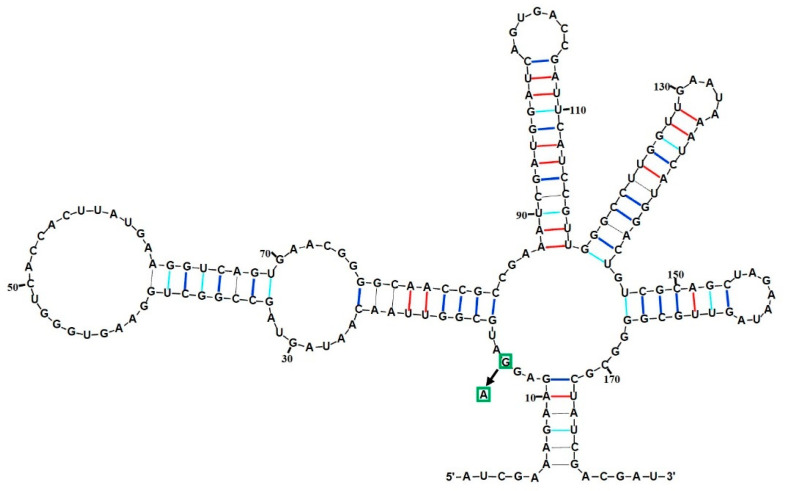
Predicted secondary structure of *Lb. plantarum* lysine riboswitch. All the secondary bonds were shown in color. One point mutation (G14A) is indicated with a green box.

Next, the genome sequences of *Lb. plantarum* WCFS1 was predicted using a Riboswitch Scanner to find out whether a native lysine riboswitch existed. As shown in Table S4, only one lysine riboswitch located in the upstream region of the lysine transport protein (*lysP* gene, lp1008) was detected. The secondary structure was predicted by uploading the sequences of putative lysine riboswitch onto RNAcentral (https://rnacentral.org/, accessed on 12 March 2022). As shown in Figure 2, a typical lysine-sensing-box motif was determined. The secondary bonds were colored and labeled using RNAcanvas. Thus, the newly found lysine riboswitch was designated as LPRS and amplified from *Lb. plantarum* WCFS1 for the following experiments.

### 3.2. Construction of Lactobacillus Lysine Riboswitch-RFP Sensor

**Figure 3:**
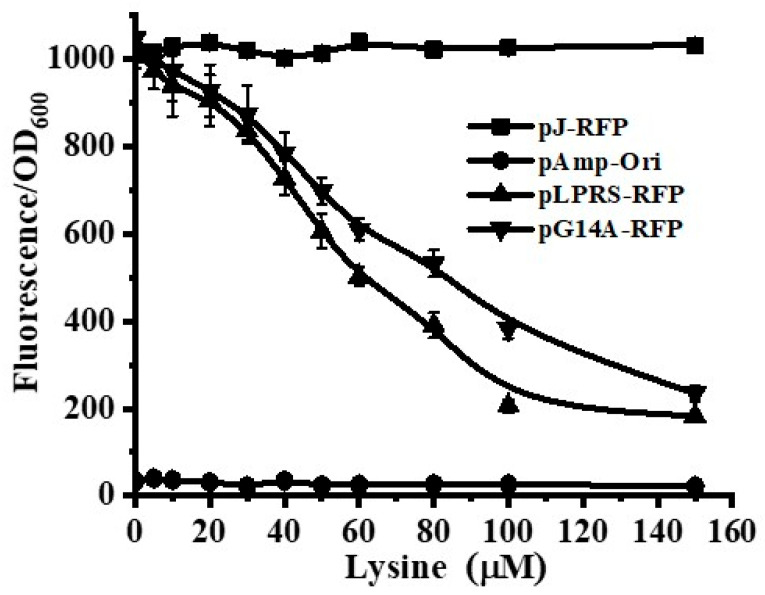
Dose-response curve of the RFP sensor over a wide range of lysine concentrations. Plasmid pJ-RFP was used as the positive control and plasmid pAmp-Ori as the negative control. Plasmid pLPRS-RFP was constructed by fusing *Lb. plantarum* wild-type lysine riboswitch-RFP. Plasmid pG14A-RFP was introduced as one point mutation in the lysine riboswitch. Error bars represent the standard deviation from three replicates.

To determine the potential of the putative lysine riboswitch of *Lb. plantarum* WCFS1, one biosensor with a fluorescent reporter gene (*rfp*) was constructed. First, the constitutive promoter BBA_J23100 and LPRS were amplified and then fused with pJ-RFP to create the vector pLPRS-RFP, in which the fluorescence protein was controlled by LPRS. To estimate the efficiency of the riboswitch, plasmid pLPRS-RFP was transformed into DH5α cells. Previous work demonstrated that lysine riboswitches were quite sensitive to lysine concentration. To ensure that the lysine riboswitch-RFP sensor was strictly related to lysine, the following experiments were performed at lysine concentrations of less than 100 μM. *E. coli* DH26 was cultured in RDM-Amp with various lysine concentrations (0, 5, 10, 20, 30, 40, 50, 60, 80, 100, and 150 μM). The pJ-RFP was used as a positive control and the pAmp-Ori as a negative control. As depicted in Figure 3, the dose response of the lysine riboswitch-based fluorescence sensor was characterized. As expected, the putative lysine riboswitch of *Lb. plantarum* functioned as a repressor to reduce gene expression. Importantly, the relative fluorescence of pLPRS-RFP was substantially repressed when the lysine concentration was increased from 10 μM to 100 μM, suggesting that the efficiency of LPRS was much higher than that of *E. coli* lysine riboswitch [23]. To confirm that the repression was due to lysine binding, a mutation in the aptamer of *Lb. plantarum* lysine riboswitch was introduced by using a site-directed mutagenesis kit (Vazyme). As expected, a single-point mutation (pG14A-RFP), which could release the repression induced by lysine binding in the aptamer region, resulted in a marked reduction of the relative repression (Figure 3). Thus, this lysine-dependent repression proved that the lysine-binding in the aptamer of *Lb. plantarum* lysine riboswitch was required for regulating gene expression.

### 3.3. Selection of Engineered Lysine Riboswitches with Optimized Properties

Although the natural *Lb. plantarum* lysine riboswitch could repress gene expression, but it was still attractive to develop more engineered lysine riboswitches, which would be used for the increasing demand for the regulation of gene expression. Regarding this, the construct pLPRS-tetA-RFP generated from pLPRS-RFP was applied to create a lysine riboswitch library using degenerate primers. The degenerate random bases were inserted into the region between the aptamer and the ribosome binding sites (RBS) by PCR. After the *E. coli* transformation, the library was prepared for colony screening. After two rounds of dual genetic selection, 31 colonies were activated by lysine (Lys-A). Meanwhile, 56 colonies were repressed by lysine (Lys-R). Ten riboswitch colonies from each group were sent for sequencing. The randomized sequences of each riboswitch colony are detailed in Table S5. 

**Figure 4:**
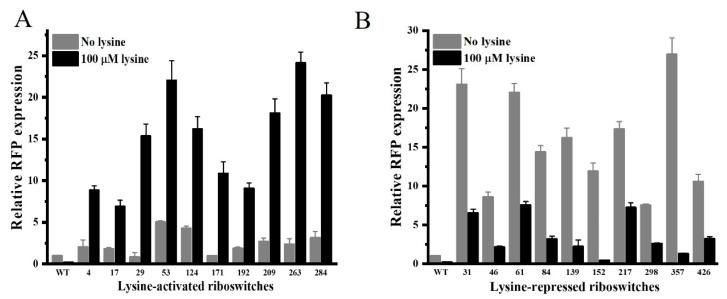
Relative RFP expression of the natural *Lb. plantarum* lysine riboswitch (LPRS, WT), the selected lysine-activated riboswitch clones (**A**) and lysine-repressed riboswitch clones (**B**) measured in the absence and presence of 100 μM lysine.

After being sequenced, the validated Lys-A riboswitches were first retransformed back into *E. coli* DH5α cells and then cultivated in RDM-Amp supplemented with or without 100 μM lysine. As depicted in Figure 4A, the relative RFP fluorescence of the tested Lys-A riboswitch colonies was promoted by the additional 100 μM lysine. Especially among all the selected engineered riboswitches, the RFP expression of Lys-A263 activated by lysine was the highest (>12-fold) compared to that of uninduced culture. On the other hand, RFP expression of the tested Lys-R riboswitch colonies was repressed in the presence of lysine (Figure 4B). Importantly, Lys-R152 was found to have the strongest repressive efficiency in the presence of lysine (>28-fold). Thus, due to their relatively optimized properties, the engineered Lys-A263 and Lys-R152 were chosen for the following work, respectively.

### 3.4. Construction of a Riboswitch-Based Mutant in C. glutamicum

**Figure 5:**
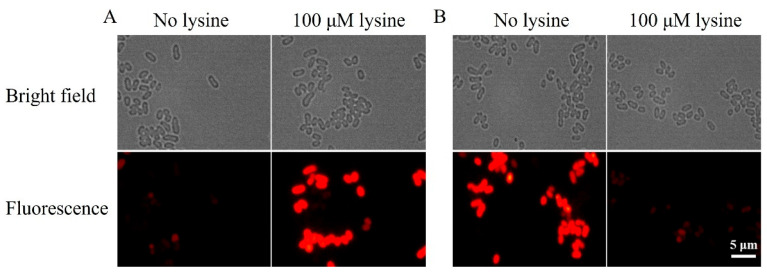
Fluorescence microscopy images of *C. glutamicum* QW36 (**A**) and QW37 (**B**) incubated in a medium without lysine (left panels) or with 100 μM lysine (right panels). Scale bar: 5 μm.

To test whether *Lb. plantarum* lysine riboswitches were functional in *C. glutamicum*, the selected engineered riboswitches were first fused with RFP and then inserted into expression vector pEC-XK99E to generate pEC-A263-RFP and pEC-R152-RFP, respectively. After retransformed into *C. glutamicum* ATCC 13032, the cells were incubated with or without lysine in DMM. After cultivation, the cells were observed by fluorescence microscopy (Leica DM4 B Upright Microscopes, Leica, Wetzlar, Germany). As expected, the strain QW36 harboring pEC-A263-RFP showed a strong level of RFP expression in the presence of 100 μM lysine compared to the cells cultured in the absence of lysine (Figure 5A). This suggested that Lys-A263 could be activated by lysine in *C. glutamicum*. On the other hand, a substantial repression in fluorescence was observed when the strain QW37 harboring pEC-R152-RFP was cultured with 100 μM lysine compared to the cells incubated without lysine, implying R152 could repress gene expression in the presence of lysine (Figure 5B). Altogether, these results proved that the selected engineered lysine-activated and repressed riboswitches could be applied to regulate gene expression in *C. glutamicum*. 

The selected engineered lysine riboswitches were then introduced into lysine-producing *C. glutamicum* QW45, in which endogenous lysine could be used as a signal to control gene expression. First, the plasmid pA263-lysC was constructed by fusing Lys-A263 with the *lysC* gene. After homologous recombination, Lys-A263 was expected to replace the regulatory region between the native promoter of *lysC* and the ORF of the *lysC* gene. Then, the mutant QW48 was screened by PCR and subsequently determined by sequencing. The same procedure was also carried out on QW45 to generate the mutant QW53 by using pR152-hom [23], in which the threonine biosynthesis was controlled by Lys-R#152.

Interestingly, the growth of QW48 was similar to that of QW45 in DMM; however, QW53 could not grow in DMM. Further, the specific activities of AKIII and HSD at the early exponential phase were measured in DMM, respectively. It was found that the specific activity of AKIII in QW48 was higher than that in the parent strain QW45 (Table 1). Unexpectedly, the specific activity of HSD of QW53 was much lower, indicating that the expression of the *hom* gene was greatly repressed by Lys-R152. This result suggested that the up-regulated expression of the *lysC* gene would not have imbalanced the metabolic flux of cell growth. Meanwhile, the downregulation of the *hom* gene reduced the biosynthesis of the endogenous homoserine, resulting in the inhibition of cell growth at the beginning. Importantly, it was speculated that the repression efficiency of Lys-R152 was significant, indicating that the basal expression of the *hom* gene was not sufficient for supporting cell growth. After carefully comparing the relative expression of wide-type LPRS and other engineered lysine-repressed riboswitches (Figure 4B), another lysine-repressed riboswitch Lys-R357 which exhibited both a higher basal expression and stronger repression was selected as an alternative to repress *hom* gene expression. Therefore, a new mutant QW54 was constructed by using Lys-R357 to repress the *hom* gene. After being tested in DMM, it was found that the cell growth of QW54 was slightly delayed compared to that of QW45. Finally, an engineered dual-gene mutant QW55 was created, in which *lysC* and *hom* were under the control of Lys-A263 and Lys-R357, respectively.

### 3.5. Lysine Production Enhanced by Riboswitch-Controlled Mutants

**Figure 6:**
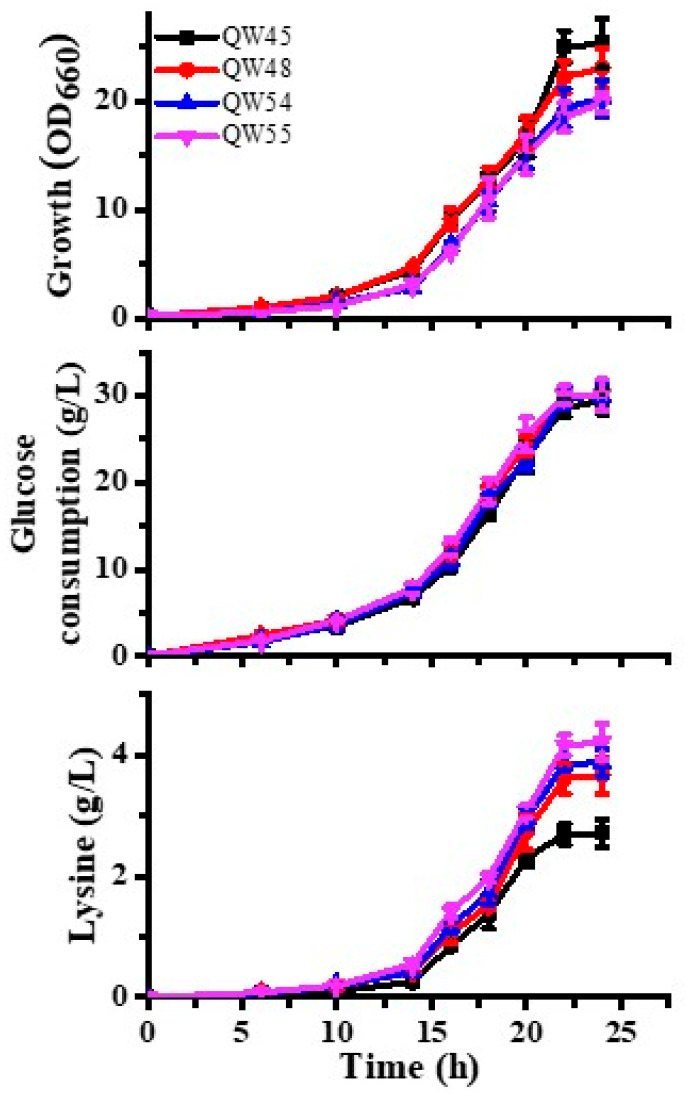
Fermentation results with *C. glutamicum* QW45, QW48, QW54, and QW55 in shake flasks. The data represent mean values and standard deviations from three independent measurements. OD_660_ means the optical density at 660 nm.

To assess the efficiency of engineered riboswitch mutants, lysine productions were examined in batch processes. In batch fermentations, the cell growth of the A263-*lysC* mutant QW48 was similar to that of the control strain QW45 in DMM, indicating that the up-induced expression of AKIII would not disturb the cell growth. Meanwhile, the R357-*hom* mutant QW54 and double-site mutant QW55 both showed slightly delayed growth, indicating that although the biosynthesis of intracellular homoserine was repressed, it was still sufficient for growth during the whole process (Figure 6). Furthermore, the glucose consumption of the four strains was approximately identical in the end. In 24 h, the yield of lysine in mutant QW45 was 0.113 ± 0.009 mol per mol of glucose. Additionally, the yield of mutant QW48 was 0.15 ± 0.012 mol per mol of glucose, which was 35% higher than that of the strain QW45. These results proved that the key gene expression of lysine biosynthesis could be effectively activated by an engineered lysine riboswitch, resulting in increased lysine production.

On the other hand, as shown in Figure 6, it could be seen that the growth of QW54 was delayed at the beginning due to the reduction of HSD. After 24 h, the OD values of QW54 declined, indicating that the number of cultivated cells was restricted by the limited intracellular homoserine. The final values of optical density of QW54 were lower than that of QW45, suggesting that the serious reduction of growth in the mutant was due to the inhibition of riboswitch-controlled HSD. Interestingly, the rate of glucose consumption of QW54 was even slightly higher than that of QW45. The final yields of lysine in QW54 reached 0.159 mol per mol of glucose in 24 h (Table 1), which was 43% higher than that of the strain QW45. The results proved that the increased lysine was apparently due to the inhibition of the biosynthesis of threonine. Thus, it could be strongly postulated that the flux was mainly used for producing lysine by repressing the expression of the *hom* gene. 

After the single-gene mutant was tested, the dual-gene mutant QW55 was further characterized by batch fermentation. From Figure 6, the growth of QW55 was identical to that of QW54. However, the rate of glucose consumption of QW55 was even higher than that of QW54, suggesting that the glucose utilization was more effective. Importantly, the final yields of lysine in QW55 reached 0.173 mol per mol of glucose, which was 53% higher than that of QW45 (Table 1). On the basis of these findings, it was concluded that the engineered Lys-A and Lys-R riboswitches were both functional in the same mutant. These results also indicated that the selected engineered lysine riboswitches could simultaneously control two different metabolic pathways. 

## 4. Discussion

There is an increasing interest in applying riboswitches for many different purposes [2]. Riboswitches have been commonly used as effective tools to regulate gene expression. In this study, we demonstrated that *Lb. plantarum* lysine riboswitch could be used as a powerful genetic tool to significantly enhance lysine production in *C. glutamicum*. Therefore, riboswitches provide an alternative, feasible strategy for metabolic engineering.

In this study, the functionality of the natural lysine riboswitch of *Lb. plantarum* was first examined by constructing a riboswitch-RFP sensor. Previous studies have demonstrated the attractive applications of riboswitch-based sensors. A natural coenzyme B_12_ riboswitch was demonstrated to regulate vitamin B_12_ biosynthesis [38]. Furthermore, several artificial riboswitches were used for detecting specific target molecules [39,40]. Synthetic RNA devices, for example, lysine riboselectors could be used to facilitate strain improvement [41]. Here, natural lysine riboswitch of *Lb. plantarum* also showed a good relationship with lysine concentration. However, the natural one still needed to be optimized to be suitable for more bioprocesses, especially for industrial fermentation.

Here, the well-studied lysine riboswitch was integrated into the chromosome of *C. glutamicum* to dynamically promote *lysC* expression and simultaneously repress *hom* expression using intracellular lysine as the signal. However, the growth of the R152-hom mutant QW53 was markedly repressed in DMM. Previous work also reported that the growth of the *E. coli* lysine riboswitch-gltA mutant was accomplished but obviously slower than that of the parent strain [23]. Considering the high sensitivity of engineered lysine riboswitch, it was speculated that the different repression efficiencies might be the result of original promoters of target genes. The specific enzyme activity assay demonstrated that the biosynthesis of HSD had been repressed since the start of cell growth. The weaker constitutive promoter P*_hom_* might result in less amount of mRNA of *hom* in the cell [42]. Due to this, another Lys-R riboswitch which showed a higher basal expression of the downstream gene was selected to partially restore the cell growth. This limitation might come from the fact that the corresponding concentration of engineered lysine riboswitch was a bit narrow. In the future, lysine riboswitch should be further optimized with other genetic elements of metabolic engineering to make a balance between growth and production.

In this study, we introduced engineered lysine riboswitches to promote biosynthetic pathways and also repress competitive by-pathways. As illustrated in Figure 6, higher productivities of the riboswitch-controlled mutants were achieved in fermentations. Due to the activation of AKIII and also the inhibition of HSD during the process, more flux was predicted for lysine biosynthesis, resulting in higher lysine production. However, the yield of lysine of LPECRS#16-lysE was even higher than that of QW55. The most likely cause of this difference was the target genes which were under the control of riboswitches. Further, it was still concluded that the riboswitches from different sources combined with promoters might result in different efficiencies of lysine production. 

A major advantage of lysine riboswitch-controlled enzyme is the mechanism of its function. As known, cells have to go through from DNA to RNA to protein during the whole growth in promoter exchange or protein engineering systems, which may be not so economical for cell processes. Here, we used a riboswitch to control RNA translation. The biosynthesis of protein is activated or repressed by an intracellular signal, which can accelerate product production. In addition, the exogenous molecules may be drawn into the cell to activate or inhibit the promoters, which may result in unexpected side effects. In our study, lysine is not only the final product but also works as the signal molecule to bind the lysine riboswitch to activate or repress gene expression. Protein engineering is also a powerful tool in metabolic engineering [43]. However, the screening and optimizing procedures have to be conducted again from the beginning if the target protein is changed. This work has shown that both lysine and threonine biosynthesis could be simultaneously controlled by the lysine riboswitch in the same mutant. It is believed that it is easier to introduce lysine riboswitch to regulate other metabolic pathways to improve lysine production in *C. glutamicum*. 

However, there are still some challenges that remain. First of all, the corresponding concentration to lysine riboswitch was at the micromolar level, which is a limitation for further applications. Although there are successful artificial riboswitches reported, it is still difficult to optimize natural riboswitches. Additionally, natural lysine riboswitch was used to repress the expression of target genes and thus could not be more easily used to up-regulate the expression of enzymes favored for lysine production. Therefore, it is of great interest to engineer more lysine riboswitches to facilitate the development of lysine producers.

## 5. Conclusions

Overall, the successful research of this work achieves the objective of utilizing Lactobacillus lysine riboswitch to dynamically control metabolic pathways for lysine production. First, a natural Lb. plantarum lysine riboswitch (LPRS) was determined and demonstrated to function as a repressor to down-regulate gene expression. Then, engineered lysine-activated/repressed riboswitches were successfully generated and utilized to regulate the key gene expressions of metabolic pathways in *C. glutamicum*. Importantly, the two-way regulative combination of engineered riboswitches efficiently increased the production of lysine from 0.113 mol/mol glucose to 0.173 mol/mol glucose in batch fermentation, about a 53% improvement. Our work provides a theoretical basis for the application of *Lb. plantarum* lysine riboswitches in metabolic engineering. Meanwhile, we anticipate that other natural and also engineered riboswitches can be developed to improve the performance of industrial microorganisms. 

## Figures and Tables

**Table 1:** Fermentation: L-lysine production by *C. glutamicum* QW45 and its riboswitch derivatives.

Strain	Lysine Yield ^a^	Final Growth (OD_660_)	Specific Activity of Enzyme (μmol min^−1^ mg^−1^)	
			Aspartate Kinase (AK III)	Homoserine Dehydrogenase (HSD)
QW45	0.113 ± 0.009	25.32 ± 2.29	0.837 ± 0.064	0.915 ± 0.023
QW48	0.15 ± 0.012	23.07 ± 1.82	0.973 ± 0.004	/
QW54	0.159 ± 0.008	20.24 ± 1.66	/	0.782 ± 0.036
QW55	0.173 ± 0.011	19.86 ± 0.85	0.919 ± 0.015	0.803 ± 0.022

^a^ Lysine yield: mol lysine per mol glucose consumed. The mean values were generated from three independent experiments. /: No detection.

## Data Availability

The datasets presented in this study are available on request to the corresponding author.

## Supplementary Materials

The following supporting information can be downloaded at: https://www.mdpi.com/article/10.3390/microorganisms12030606/s1, Table S1: Bacterial strains and plasmids used in this study; Table S2: Primers used in this study; Table S3: Predicted results of lysine riboswitch in *C. glutamicum* ATCC 13032 using Riboswitch Scanner; Table S4: Predicted results of lysine riboswitch in *Lb. plantarum* WCFS1 13032 using Riboswitch Scanner; Table S5: The sequences of the engineered *Lb. plantarum* lysine riboswitches.
