# Supplementary material for: Engineering a Lactobacillus Lysine Riboswitch to Dynamically Control Metabolic Pathways for Lysine Production in Corynebacterium glutamicum
Source: Microorganisms. 2024 Mar 18;12(3):606. doi: 10.3390/microorganisms12030606 (PMC10974012; doi:10.3390/microorganisms12030606)
Supplement: Supplementary file 1 [file microorganisms-12-00606-s001.zip › microorganisms-2777699-supplementary.pdf]

Supplementary Information

Table S1. Bacterial strains and plasmids used in this study.

Strains or plasmids	Relevant characteristics[a]	Source or reference
Strains		
E. coli		
DH5α	F^- *endA1 glnV44 thi-1 recA1 relA1 gyrA96 deoR nupG purB20* φ80d*lacZ*ΔM15 Δ(*lacZYA-argF*)U169, hsdR17($r_K^- m_K^+$), λ^-	Lab collection
DH-26	DH5α pLPRS-RFP	This study
DH-31	DH5α pLPRS-tetA-RFP	This study
C. glutamicum		
ATCC 13032	Wild-type strain	Lab collection
QW36	pEC-A263-RFP, derived from ATCC 13032	This study
QW37	pEC-R152-RFP, derived from ATCC 13032	This study
QW45	*lysC* (Q298G), derived from ATCC 13032	This study
QW48	LPRS-A263-lysC, derived from QW45	This study
QW53	LPRS-R152-hom, derived from QW45	This study
QW54	LPRS-R357-hom, derived from QW45	This study
QW55	LPRS-A263-lysC, LPRS-R357-hom, derived from QW45	This study
Plasmids		
pAmp-Ori	Cloning vector	Lab collection
pJ-RFP	Expression vector	Lab collection
pK18mobsacB	Kan^R, SacB	Lab collection
pEC-XK99E	Expression vector	Lab collection
pLPRS-RFP	BBa_J23100, *Lb. plantarum* lysine riboswitch (LPRS), RFP	This study
pG14A-RFP	LPRS (G14A), derived from pLPRS-RFP	This study
pLPRS-tetA-RFP	tetA, derived from pLPRS-RFP	This study
pEC-A263-RFP	LPRS-A263 fused with *RFP* gene, derived from pEC-XK99E	This study
pEC-R152-RFP	LPRS-R152 fused with *RFP* gene, derived from pEC-XK99E	This study
pA263-lysC	LPRS-A263 fused with *lysC* gene, derived from pK18mobsacB	This study
pR152-hom	LPRS-R152 fused with *hom* gene, derived from pK18mobsacB	This study
pR357-hom	LPRS-R357 fused with *hom* gene, derived from pK18mobsacB	This study

Table S2. Primers used in this study.

Primer	Sequence	Description
LPRS-F	atcgaaagaagaggatgcg	For *Lb. plantarum* lysine riboswitch amplification
LPRS-R	ctgccattgcagaacctcc	
RFP-F	atggcttcctccgaagac	For *RFP* amplification
RFP-R	ttaagcaccggtggagtgac	
BR-F	agtagctagcactgtacctag	For backbone of pJ-RFP amplification
BR-R	gatgctgaaaagaacgccg	
tetA-F	atgcaagtcgacctgctgg	For *tetA* amplification
tetA-R	cctcctccagatcctcctccagatccacctccaagggtcgaggtggcccggc	
RSlib-F	NNNNNNNNNNNNNNNNNNNNgaaatcatgctgaaaagaacg	For pLPRS-tetA-RFP amplification
RSlib-R	NNNNNNNNNNNNNNNNNNNNcctcttctttcgatatactcaac	
lysC-F	aggtggccctggtcgtacag	For *lysC* amplification
lysC-R	ctttagcgtccggtgcctgc	
hom-F	atcatgacctcagcatctgc	For *hom* amplification
hom-R	attagtccctttcgaggcgg	
K18-F	GGATCCCCTGACACGAGGTAGTTatgacctcagcatctgccccaag	For backbone of pK18mobsacB amplification
K18-R	GGCCTGCGCTAGCGCAGGTAGTACctattactttgtttcggccaccc	

Table S3. Predicted results of lysine riboswitch in *C. glutamicum* ATCC 13032 using Riboswitch Scanner.

Genome_ID	Genome_Name	Taxonomy_name		Riboswitch Location	Riboswitch Strand	Riboswitch class	Downstrea[illegible] gene location
NC_003450.3	Corynebacterium glutamicum ATCC 13032	Actinobacteria	201174	66279-66442	-	FMN	65504-66197
NC_003450.3	Corynebacterium glutamicum ATCC 13032	Actinobacteria	201174	554235-554393	-	cobalamin	552944-554129
NC_003450.3	Corynebacterium glutamicum ATCC 13032	Actinobacteria	201174	741074-741181	-	TPP	740224-741016
NC_003450.3	Corynebacterium glutamicum ATCC 13032	Actinobacteria	201174	868388-868556	-	ydaO-yuaA	867799-868381
NC_003450.3	Corynebacterium glutamicum ATCC 13032	Actinobacteria	201174	1126306-1126415	+	TPP	1127012-1128353
NC_003450.3	Corynebacterium glutamicum ATCC 13032	Actinobacteria	201174	1371634-1371746	-	TPP	1369550-1369877
NC_003450.3	Corynebacterium glutamicum ATCC 13032	Actinobacteria	201174	1372540-1372656	+	SAM-IV	1372804-1373134
NC_003450.3	Corynebacterium glutamicum ATCC 13032	Actinobacteria	201174	1542916-1543023	-	TPP	1539816-1542108
NC_003450.3	Corynebacterium glutamicum ATCC 13032	Actinobacteria	201174	2150221-2150334	+	TPP	2150334-2151000
NC_003450.3	Corynebacterium glutamicum ATCC 13032	Actinobacteria	201174	2320608-2320796	-	ydaO-yuaA	2319965-2320595

Table S4. Predicted results of lysine riboswitch in *Lb. plantarum* WCFS1 13032 using Riboswitch Scanner.

Genome_ID	Genome_Name	Taxonomy_name		Riboswitch Location	Riboswitch Strand	Riboswitch class	Downstream gene location
NC_004567.2	Lactobacillus plantarum WCFS1	Firmicutes	1239	99249-99354	+	TPP	99432-100224
NC_004567.2	Lactobacillus plantarum WCFS1	Firmicutes	1239	202607-202700	-	TPP	201934-202504
NC_004567.2	Lactobacillus plantarum WCFS1	Firmicutes	1239	482228-482273	+	PreQ1	482281-482788
NC_004567.2	Lactobacillus plantarum WCFS1	Firmicutes	1239	761448-761599	+	glmS	761631-763449
NC_004567.2	Lactobacillus plantarum WCFS1	Firmicutes	1239	928916-929097	+	lysine	929240-930692
NC_004567.2	Lactobacillus plantarum WCFS1	Firmicutes	1239	1189828-1189933	+	SMK_box	1191254-1192757
NC_004567.2	Lactobacillus plantarum WCFS1	Firmicutes	1239	1705901-1706022	-	FMN	1705192-1705777
NC_004567.2	Lactobacillus plantarum WCFS1	Firmicutes	1239	1741167-1741232	-	fluoride	1739825-1741115

NC_004567.2	Lactobacillus plantarum WCFS1	Firmicutes	1239	1874486-1874649	-	ykoK	1872642-1874385
NC_004567.2	Lactobacillus plantarum WCFS1	Firmicutes	1239	2410476-2410574	+	purine	2410633-2411974
NC_004567.2	Lactobacillus plantarum WCFS1	Firmicutes	1239	2519515-2519559	-	PreQ1	2518513-2519494
NC_004567.2	Lactobacillus plantarum WCFS1	Firmicutes	1239	2968731-2968829	-	purine	2966961-2968653

Table S5. The sequences of the engineered *Lb. plantarum* lysine riboswitches.

Engineered LPRS clone	Sequence
Lysine-activated	
4	cccgtgagacggtcgttagcaccc
17	gtgagcgaacatatgatagcatcgg
29	gtacaccgcacaccagcagcttg
53	ggtgtgacacggttcacc
124	gaggccggcacagagtgtctcg
171	tcgcgtctggtgattgatgtgctctg
192	gaccggcgtaagctcgagatagtg
209	cggttgagcggtgtgtatatgcaag
263	acgatacgtcgcgcatgtgga
284	gctggaccgctccatctgcggac
Lysine-repressed	
31	cgctcaggtctccgatgagtcg
46	agggtcgtactcgcattcgg
61	tctacgtgaagcattctggtaagatg
84	gaccacatgcgatgactcagcag
139	ctcccgaatgattgcaatagg
152	tgactggtctatcaggctgtacg
217	ccgcatcgccgtaggtggcag
298	catcagccagttggcttgtcggt
314	aggcacgcaccgttagcgcgcg
352	tccagtgcagtgttggagatatgtc
